# Home Health Care Providers’ Readiness to Care for Children and Youth With Complex Medical Conditions: Protocol for a Scoping Review

**DOI:** 10.2196/76796

**Published:** 2025-07-04

**Authors:** Joanne Tay, Margaret Saari, Adam Mulcaster, Edward Cruz

**Affiliations:** 1 University of Windsor Windsor, ON Canada; 2 Saint Elizabeth Research Centre Toronto, ON Canada

**Keywords:** children, home care, practice readiness, protocol, scoping review

## Abstract

**Background:**

Children and youth with medical complexities have chronic conditions, functional limitations, and extensive care needs requiring significant family involvement and frequent health service use. Pediatric medicine has improved their life expectancy, shifting care from acute to home settings. In Ontario, home care is publicly funded and includes nursing, therapy, personal support, and allied health services. However, families continue providing the bulk of care, often at great personal costs. As care complexity increases, so does the reliance on home care providers. Yet, many providers report feeling unprepared due to insufficient pediatric training, lack of supervision, and system-level gaps. Here we applied Weiner’s theory of organizational readiness for change, framing readiness as a shared psychological state involving commitment to and confidence in delivering change, thus helping conceptualize provider readiness as encompassing individual competence, contextual supports, and motivational factors. Despite its importance, provider readiness in pediatric home care remains poorly understood.

**Objective:**

We examine how home care providers perceive their readiness to care for children and youth with medical complexities, factors influencing readiness, and strategies proposed to enhance their capacity to deliver safe, coordinated, developmentally appropriate home care.

**Methods:**

This scoping review follows the updated Joanna Briggs Institute scoping review methodology and PRISMA-ScR (Preferred Reporting Items for Systematic Reviews and Meta-Analyses extension for Scoping Reviews) guidelines. Articles focusing on (1) children and youth with medical complexities aged 0-18 years receiving home care and (2) with at least one chronic medical condition, and (3) paid home care providers will be searched on MEDLINE, CINAHL, Scopus, ProQuest Nursing & Allied Health, PsycINFO, Cochrane Central Register of Controlled Trials, and Cochrane Database of Systematic Reviews. Gray literature will be searched on Google Scholar and ProQuest (Dissertations & Theses). A 2-stage screening process will be conducted in Covidence, involving title and abstract screening and full-text review by 2 independent reviewers. Data will be extracted using predefined charting categories and analyzed using both qualitative and quantitative approaches.

**Results:**

As of May 2025, screening is underway. The review will map key themes across provider types, care settings, and training contexts and identify factors influencing provider readiness including access to training, system navigation supports, and workplace role clarity and chart recommendations or strategies to enhance readiness. Gaps in pediatric-specific competencies and workforce preparation may also emerge.

**Conclusions:**

This review will synthesize current evidence on provider readiness in pediatric home care, identifying strengths, challenges, and development areas. Findings will inform provider training, workforce development, and policy strategies to ensure children and youth with medical complexities receive safe, coordinated, effective home care. A deeper understanding of provider readiness will support more sustainable home care systems and improve outcomes for children and youth with medical complexities and their families.

**Trial Registration:**

OSF Registries 10.17605/OSF.IO/N4FAJ; https://osf.io/n4faj

**International Registered Report Identifier (IRRID):**

PRR1-10.2196/76796

## Introduction

Children and youth with medical complexities are a diverse group of individuals who share the following characteristics: (1) they have chronic and severe conditions, (2) they have functional limitations, (3) they have extensive family-identified needs, and (4) they require substantial use of health care services [1,2]. Diagnoses common to them are numerous and include preterm birth, as well as congenital genetic, metabolic, and neurological conditions that require constant and close monitoring of their physical, emotional, behavioral, and developmental well-being [3]. While many children and youth with medical complexities are at risk of premature mortality, advances in medical care and technology have extended life expectancy for others [4,5].

Care for children and youth with medical complexities was previously delivered almost exclusively in acute care settings by specialized health care providers such as pediatricians and pediatric nurses. However, shifts in family preferences to receive care closer to home and rising health care costs have moved much of this care to home and community care settings [6-8]. In regions such as Ontario, Canada, home care services are funded and organized through the provincial government, specifically Ontario Health atHome [9]. Home care services frequently accessed by children and youth with medical complexities include but are not limited to nursing, physiotherapy, occupational therapy, dietetics, speech therapy, social work, and personal support worker care, each playing an essential role in supporting children and youth with medical complexities and relieving the caregiving burden on their families. However, families continue to spend 15.7 million hours per year in caring for children with acute, chronic, or complex health issues [10].

As the medical complexity of the child increases, the required caregiving hours also rise. For example, it takes approximately 130 hours to care for a child with attention-deficit issues and 629 hours to care for a child with complex medical conditions [10]. Many parents willingly take on these responsibilities but report significant impacts on self-care, couple time with their partners, and parenting time and opportunity with their other children [11]. While this review focuses on provider readiness, these caregiver experiences highlight the consequences of inadequate or inconsistent home care support and reinforce the importance of a well-prepared provider workforce. Without timely and adequate support from home care services, prolonged caregiving puts parents at heightened risk of burnout and other mental health conditions, such as anxiety and depression [12,13].

Despite its growing importance, Ontario’s pediatric home health care services are notably fragmented and complicated to navigate [14,15]. A 2020 Canadian Institute of Health Information report identified 97,561 children and youth with medical complexities across Canada; only 43% of this population received home care [16]. Families frequently describe the system as slow, reactive, and challenging to access, requiring them to repeatedly “fight” for supplies or services [11,17,18]. Maynard et al [8] found that 7 in 10 children experienced an additional 53.9 hospitalization days due to the lack of home care nursing services. Parents report that home care services are not always guaranteed, necessitating private nursing care while waiting for publicly funded home care services [19]. The consequences of inadequate home care are significant: limited availability of home health care nursing and insufficient support are linked to extended hospital stays and avoidable readmissions [8,20].

Caring for children and youth with medical complexities at home requires home care providers who are both competent in pediatric care and confident in managing the child’s specific clinical needs [21,22]. Their needs may include the use of medical devices, be on multiple medications, and require other supports at home and in school due to their functional limitation [16]. Given the complexity of care, McCann [18] reported that parents “do not trust respite care providers to provide a suitable standard of care for their child.” Research has highlighted that many home health care providers feel unprepared to provide specialized pediatric care due to a lack of specialized training opportunities to gain the necessary experience and skills, a lack of professional practice support from home care organizations, and limited guidance from supervisors [6]. This review, therefore, draws conceptually on Weiner’s theory of organizational readiness for change, which frames readiness as a shared psychological state encompassing commitment to and confidence in implementing a change [23]. In our context, readiness is understood as a multidimensional construct comprising individual competence (eg, knowledge, skills, and confidence), contextual supports (eg, training, supervision, and resources), and motivational factors (eg, perceived role clarity and value alignment). This framework supports a nuanced examination of both system-level and individual factors that shape provider preparedness to care for children and youth with medical complexities at home [23]. While individual studies have examined provider experiences and training needs, there is limited synthesis on the overall readiness of the home care workforce to deliver safe, developmentally appropriate care to children and youth with medical complexities [16,24,25]. In particular, few studies have explored readiness across provider types or contextual factors influencing preparedness, especially within the Canadian health care system [19,26].

This gap in providers’ readiness leaves many children and youth with medical complexities with substantially unmet needs that parents/caregivers often have to manage independently [27]. Although studies suggest that home is the preferred setting for the long-term care of children and youth with medical complexities, the reported lack of provider preparedness presents a serious safety concern. It also affects families’ confidence and decision to entrust their children and youth with medical complexities to home health care professionals [3,28,29]. The continued lack of timely and adequate home health care places parents and/or caregivers at risk of burnout, emotional and psychological distress, financial demands, and social isolation [12]. These outcomes, while not the primary focus of this review, underscore how provider readiness directly influences the sustainability of family caregiving. In this context, understanding the factors influencing provider readiness and identifying strategies to support and strengthen it are critical for improving outcomes for children and youth with medical complexities and their caregivers. This review does not aim to examine caregiver burden directly, but rather to explore provider-related factors that may impact quality and indirectly shape family caregiving experiences.

Therefore, it is necessary to examine the extent and nature of the literature on home and community care health care providers’ readiness to care for children and youth with medical complexities. Although this review focuses primarily on provider-related factors, it also considers the characteristics of children and youth with medical complexities receiving home care to better contextualize provider readiness and the types of support required in different care contexts. Findings from this review will help inform policy, workforce development, and future research aimed at strengthening the capacity of home care providers to meet the needs of children and youth with medical complexities and their families.

The research team, including 2 nursing faculty members (JT and EC), 1 nursing librarian (AM), and 1 clinical scientist (MS), developed the overarching review question, “What is known about home health care providers’ readiness to care for children and youth with medical complexities at home?” The specific questions are as follows:

- What are the characteristics of children receiving home health care, and how might these shape provider readiness?
- How do home health care nurses, including registered nurses, practical nurses, and other professionals such as therapists and personal support workers, perceive their readiness to care for children and youth with medical complexities at home?
- What factors influence the readiness of home health care providers to care for children and youth with medical complexities?
- What recommendations or strategies have been proposed to enhance providers’ readiness in pediatric home care?

## Methods

The proposed scoping review will be conducted in accordance with the JBI (Joanna Briggs Institute) methodology for scoping reviews, and in line with the PRISMA-ScR (Preferred Reporting Items for Systematic Reviews and Meta-Analyses extension for Scoping Reviews) framework [28,29]. This protocol has been registered in the OSF Registries (osf.io/m58zj/). As per the JBIs’ recommendation, a critical appraisal will not be assessed because the purpose of a scoping review is to describe the extent of available evidence in the field [28].

We will include studies that examine the readiness of home care providers (eg, registered nurses, registered/licensed practical nurses, physicians, therapists, and personal support workers) who deliver care to children and youth with medical complexities aged 0 to 18 years with at least one complex medical condition (eg, epilepsy, global developmental delay, cardiopulmonary disease, neurological impairment, genetic and metabolic conditions, etc). For this review, studies must specify provider roles and focus on paid home care services to role-specific relevance. The search strategy for the full range of provider terms considered is outlined in Table 1. Readiness is defined as a combination of clinical knowledge, technical skills, critical thinking, communication, professionalism, and management of responsibilities [26,27]. Complex medical conditions are defined as individuals who (1) have chronic conditions that limit their functional ability, (2) require round-the-clock care, and (3) require substantial use of health care services. The context includes home care settings in which paid health care providers support children and youth with medical complexities through daily living assistance or more specialized interventions such as home-based antibiotic administration. Articles will be included if they report on the level of readiness among home care providers. Studies focused exclusively on non–home care settings, such as acute care, children’s emergency rooms, children’s treatment centers, schools, and respite care facilities, will be excluded unless they specifically report and discuss findings relevant to home care. 

**Table 1:** Search strategy draft for MEDLINE (Ovid).

Number	Search queries
1	(nurs* or RN* or RPN* or LPN* or NP* or APN* or APRN* or CNS* or physician* or doctor* or MD* or physiotherapist* or “speech language therapist*” or “speech-language therapist*” or “speech language pathologist*” or “speech-language pathologist*” or dietitian* or dietician* or “occupational therapist*” or OCT* or “social worker*” or “care coordinator*” or “healthcare provider*” or “health care provider*” or HCW* or “healthcare worker*” or “health care worker*” or “interdisciplinary team*” or “multidisciplinary team*”).ti,ab,kw.
2	exp Health Personnel/ or exp patient care management/
3	1 or 2
4	(pediatric* or paediatric* or peds or paeds or teen* or child* or adolescen* or youth* or infant* or newborn* or neonat* or kid* or “young* adult*” or ((one or two or three or four or five or six or seven or eight or nine or ten or eleven or twelve or thirteen or fourteen or fifteen or sixteen or seventeen or eighteen or “1” or “2” or “3” or “4” or “5” or “6” or “7” or “8” or “9” or “10” or “11” or “12” or “13” or “14” or “15” or “16” or “17” or “18”) adj1 (“year* old*” or “month* old*”))).ti,ab,kw.
5	exp Pediatrics/ or adolescent/ or young adult/ or exp child/ or exp infant/
6	4 or 5
7	(“complex care” or CMC* or CSHCN* or CYMC* or “medically complex” or “medical complexity” or “medical complexities” or “medically fragile” or life-limit* or “life limiting” or “life limited” or life-threat* or “life threatened” or “life threatening” or life-short* or “life shortening” or “life shortened” or “multiple conditions” or “chronic condition” or “chronic conditions” or “complex condition*” or “care coordination” or co-morbidit* or comorbidit* or multi-morbidit* or multimorbidit* or “technology dependence” or “technology dependent” or “high intensity care” or “neurodevelopmental disabilit*” or “neuro-developmental disabilit*” or “neurodevelopmentally disabled” or “neuro-developmentally disabled” or “global developmental delay” or GDD or “enteral nutrition” or “parenteral nutrition” or ventilat* or “mechanical ventilation” or “neurological impairment” or “neurological impairments” or “neurologically impaired” or “rare disease*” or “rare disorder*” or “rare condition*” or “genetic disease*” or “genetic disorder*” or “genetic condition*” or “special need” or “special needs” or “technology dependent” or “technology dependence” or “technologically dependent”).ti,ab,kw.
8	rare diseases/ or exp Comorbidity/ or exp neurodevelopmental disorders/ or enteral nutrition/ or Parenteral Nutrition, Home/ or exp Ventilators, Mechanical/
9	7 or 8
10	(prepare* or readiness or ready or competen* or proficien* or knowledge* or skill* or able or abilit* or confiden* or “nurs* attitude*” or “nurs* view*” or “nurs* opinion*” or “professional development”).ti,ab,kw.
11	(((home or respite or communit* or primary) adj2 (care or “health care” or healthcare or nurs*)) or ((personal or paid) adj3 caregiv*) or “personal care aide*”).ti,ab,kw.
12	home health aides/ or exp home care services/ or patient care planning/ or primary health care/ or transition to adult care/ or transitional care/ or patient navigation/
13	11 or 12
14	3 and 6 and 9 and 10 and 13
15	limit 14 to “all child (0 to 18 years)”

This scoping review will consider experimental and quasi-experimental study designs, including randomized controlled trials, nonrandomized controlled trials, before-and-after studies, and interrupted time-series studies. In addition, analytical observational studies, including prospective and retrospective cohort studies, case-control studies, and analytical cross-sectional studies, will be considered for inclusion. This review will also consider descriptive observational study designs, including case series, individual case reports, and descriptive cross-sectional studies, for inclusion. In addition, qualitative studies using methods such as phenomenology, grounded theory, ethnography, qualitative description, action research, and feminist research will be considered. In addition, any kind of systematic reviews that meet the inclusion criteria will also be considered. Gray literature, such as dissertations and theses, opinion papers, and conference presentations, will also be considered for inclusion in this scoping review. However, sources such as theoretical or conceptual papers, books, or book chapters will not be included.

The search strategy will aim to locate both published and unpublished studies. An initial limited search of MEDLINE (Ovid) and CINAHL (EBSCO) was undertaken to identify articles on the topic. The text words contained in the titles and abstracts of relevant articles and the index items used to describe the articles were used to develop a full search strategy for MEDLINE via Ovid. The search strategy, including all identified keywords and index terms, will be adapted for each included database and/or information source. The reference list of all included sources of evidence will be screened for additional studies. The databases to be searched include MEDLINE, CINAHL, Scopus, ProQuest Nursing & Allied Health, PsycINFO, Cochrane Central Register of Controlled Trials, and Cochrane Database of Systematic Reviews. Gray literature search will include dissertations and theses, opinion papers, and conference presentations. The same search strategy will be applied to ProQuest Dissertations & Theses. Papers published in any language will be included. Although the team primarily uses English as its working language, DeepL translator (DeepL) will be used for the initial translation of non–English-language articles. To ensure translation accuracy and quality appraisal, translated content will be reviewed by at least one bilingual reviewer or a native speaker familiar with the subject matter, when available. Professional translation or consultation with a language expert will be sought to validate meaning and context, where necessary.

The search strategy for this protocol was developed by author JT in consultation with a nursing librarian AM from the University of Windsor to scope out relevant studies from both published and gray literature. Keywords and search terms relating to pediatric home care and care providers were developed and adapted from published search terms. A draft search strategy for MEDLINE (Ovid) is presented in Table 1. No date restriction will be imposed on the searches, as the literature on home care for children is limited. However, studies published before 2000 will be carefully assessed for relevance, and findings will be interpreted in light of changes in pediatric care practices, health systems, and home care models over time.

Following the search, all identified citations will be collated and uploaded into Zotero (version 6.0.36; Corporation for Digital Scholarship and Roy Rosenweig Center for History and New Media), and duplicates will be removed. Potentially relevant sources will be retrieved in full, and their citation details will be imported into Covidence (Veritas Health Innovation) for screening. A 2-stage review will be conducted to screen the articles. In the first stage, 2 independent reviewers will screen articles for relevance based on the title and abstract. In the second stage, 2 reviewers (JT and EC) will perform independent full-text reviews of articles. Any disagreements among the reviewers at each stage of the selection process will be resolved with an additional reviewer (or reviewers). Reasons for excluding sources of evidence at full text that do not meet the inclusion criteria will be recorded and reported in the scoping review. Results of the screening process will be presented in a PRISMA (Preferred Reporting Items for Systematic Reviews and Meta-Analyses) 2020 flow diagram [30].

Data will be extracted from the articles included in this scoping review. A pilot test will be undertaken by 2 or more members of the review team on at least 4 articles using a data extraction tool developed by the reviewers to ensure reliability and accuracy in capturing data. After this initial review, articles will be reviewed by 2 or more independent reviewers. Any disagreements among reviewers will be resolved with additional reviewers. The extraction tool will provide an overview of knowledge and practice gaps to inform education, training, and service delivery in home and community care.

The extracted data will include specific study details, such as sample characteristics, data collection and analysis, results, and other findings. The draft data extraction tool will be refined as necessary during the data extraction process. Any refinement will be detailed in the scoping review. Findings will then be organized in a spreadsheet format. 

The PRISMA-ScR checklist will guide the reporting of the results in this scoping review. To answer review question 1, characteristics (eg, age, sex, types of diagnosis, time since diagnosis, types of medical devices, etc) will be described using frequency counts, percentages, mean, and SDs. Similarly, for review question 3, frequency counts and percentages will be used to summarize all reported influencing factors identified through quantitative data. Where available, quantitative findings related to influencing factors will also be analyzed thematically.

A qualitative descriptive approach will be used to address review questions 2 and 3. Relevant findings from included studies will be extracted and coded inductively, and thematic analysis will be conducted to identify and group key patterns in the perspectives of home care providers regarding their readiness to care for children and youth with medical complexities. For review question 4, reported strategies or recommendations to enhance provider readiness will be charted and thematically categorized. Finally, we will use different knowledge translation strategies to share results, including but not limited to open access publication in a peer-reviewed journal, presentation at home and community care conferences, and infographics to share our findings via social media such as X (formerly Twitter) and Instagram*.*

## Results

As of May 2025, data screening for the scoping review is underway. This review will identify key factors influencing provider readiness to care for children and youth with medical complexities at home, including training needs, system-level supports, and role-specific challenges. The analysis will highlight gaps in pediatric-specific competencies and workforce preparation, informing future efforts to strengthen home care capacity and provider support. Results are expected to be published in winter 2026.

## Discussion

### Anticipated Findings

This scoping review protocol addresses a critical knowledge gap in understanding provider readiness for pediatric home care. Despite the increasing prevalence of children and youth with medical complexities receiving care at home, research on provider preparedness has not kept pace with this trend [1,16]. While advances in pediatric medicine have improved survival rates and shifted care from hospital to home settings, current literature on provider readiness exists in isolation across different professional groups and care contexts, limiting understanding of broader workforce capacity issues [4-6]. This fragmentation is particularly problematic in Canada, where publicly funded home care systems vary significantly across provinces, with little comparative analysis on provider preparedness [9,14,15]. Using Weiner’s theory of organizational readiness for change as a guiding framework, this review recognizes that readiness extends beyond individual competence to encompass organizational supports and motivational factors, providing a structured lens for systematic synthesis that can inform targeted interventions [23]. While this review focuses on provider factors, the ultimate beneficiaries are children and youth with medical complexities and their families who depend on competent, confident home care providers for safe and effective care delivery [11,12]. The consequences of inadequate provider readiness extend beyond immediate care quality to affect family stress, caregiver burden, and health care usage patterns, and the review’s focus on role-specific challenges recognizes that different health care providers bring unique skills and face distinct challenges in pediatric home care contexts [8,13,21,22].

### Methodological Considerations and Strengths

The choice of scoping review methodology is particularly appropriate for this topic given the exploratory nature of the research questions and the anticipated heterogeneity in study designs, populations, and outcomes [28,29]. Unlike systematic reviews that focus on specific, well-defined questions with comparable outcomes, scoping reviews are designed to map the breadth of evidence and identify key concepts, gaps, and future research directions, which is essential when examining provider readiness where definitions, measurement approaches, and contextual factors likely vary significantly across studies [28]. The comprehensive search strategy developed in consultation with a nursing librarian ensures broad coverage of relevant databases and gray literature sources, while the inclusion of non–English language publications with translation protocols addresses potential language bias given the international scope of pediatric home care challenges [31]. Studies from 2000 onward will be included to ensure findings reflect current pediatric care practices and home care models, as earlier studies may not adequately represent the current landscape of provider roles and care delivery approaches. The 2-stage screening process with independent reviewers and conflict resolution mechanisms enhances the rigor and reliability of study selection, and the use of Covidence for screening management and pilot testing of data extraction tools further strengthens the methodological approach, with these quality measures being crucial given the anticipated complexity and heterogeneity of the literature base [30].

### Anticipated Challenges and Limitations

Several challenges are anticipated in conducting this review. The heterogeneity in how “readiness” is defined and measured across studies may complicate synthesis efforts, as provider readiness has been conceptualized variously as confidence, competence, preparedness, or capability, with measurement approaches ranging from self-report surveys to observational assessments [26,27]. Additionally, variation in health care systems, particularly between publicly funded systems like Canada’s and other models, may affect the generalizability of our findings. However, this diversity also represents an opportunity to identify universal themes and context-specific factors that influence provider readiness [9]. Finally, focusing on published literature may introduce publication bias, where unfavorable or inconclusive findings may be less likely to appear. Despite the inclusion of gray literature and translation protocols, some relevant non–English-language studies may still be missed.

### Implications for Policy and Practice

The findings from this review are expected to have significant implications for multiple stakeholder groups. For policymakers, the synthesis will provide evidence to inform workforce planning, funding allocation, and regulatory frameworks for pediatric home care, while health care organizations and home care agencies will benefit from identifying organizational factors that contribute to provider preparedness to inform quality improvement initiatives and staff development programs [32,33]. For educators and professional bodies, identifying competency gaps and training needs will inform curriculum development and continuing education programs, with an understanding of how readiness varies across health care roles, enabling the development of role-specific educational interventions and competency frameworks [34]. For researchers, this review will identify methodological approaches, outcome measures, and research gaps that can guide future investigation, with the synthesis of existing measurement approaches potentially contributing to the development of more standardized tools for assessing provider readiness in pediatric home care contexts.

### Future Research Directions

This scoping review will identify important areas for future research, including gaps in understanding specific provider types, care contexts, or populations of children and youth with medical complexities. The synthesis may reveal a need for more robust measurement tools for assessing provider readiness or longitudinal studies examining how readiness changes with experience and training over time. Additionally, the review may identify a need for intervention studies that test strategies for enhancing provider preparedness in real-world settings and implementation science studies that examine how evidence-based strategies can be effectively translated into practice across different organizational contexts. Understanding what works and how to implement effective strategies in diverse settings will be crucial for translating research findings into improved outcomes for children and youth with medical complexities.

### Conclusions

As the care of children and youth with medical complexities shifts increasingly to the home setting, understanding provider readiness becomes essential to ensuring safe, coordinated, and effective care. This scoping review will synthesize current evidence on how readiness is defined, perceived, and influenced across home care roles. Results will guide further efforts in policy, workforce training, and service planning to better equip providers and support families navigating complex home care needs.

## Abbreviations

JBI: Joanna Briggs Institute
PRISMA: Preferred Reporting Items for Systematic Reviews and Meta-Analyses
PRISMA-ScR: Preferred Reporting Items for Systematic Reviews and Meta-Analyses extension for Scoping Reviews

## References

[1] (2018). CAPHC Guideline for the Management of Medically Complex Children and Youth Through the Continuum of Care. Canadian Association of Paediatric Health Centres.

[2] Cohen E, Kuo DZ, Agrawal R, Berry JG, Bhagat SKM, Simon TD, Srivastava R (2011). Children with medical complexity: an emerging population for clinical and research initiatives. Pediatrics.

[3] Elias E, Murphy N, Council on Children with Disabilities (2012). Home care of children and youth with complex health care needs and technology dependencies. Pediatrics.

[4] Feudtner C, Hays R, Haynes G, Geyer J, Neff J, Koepsell T (2001). Deaths attributed to pediatric complex chronic conditions: national trends and implications for supportive care services. Pediatrics.

[5] Feudtner C, Christakis D, Zimmerman F, Muldoon J, Neff J, Koepsell T (2002). Characteristics of deaths occurring in children's hospitals: implications for supportive care services. Pediatrics.

[6] Foster C, Morales L, Fawcett A, Coleman C (2023). Access and quality of pediatric home healthcare: A systematic review. Home Health Care Manag Pract.

[7] Cohen E, Berry JG, Camacho X, Anderson G, Wodchis W, Guttmann A (2012). Patterns and costs of health care use of children with medical complexity. Pediatrics.

[8] Maynard R, Christensen E, Cady R, Jacob A, Ouellette Y, Podgorski H, Schiltz Brenda, Schwantes Scott, Wheeler William (2019). Home health care availability and discharge delays in children with medical complexity. Pediatrics.

[9] Ontario HA About Us. Ontario Health atHome.

[10] (2017). Inadequate home care for Ottawa-area families with sick children, study finds. CBC/Radio Canada.

[11] McCann D, Bull R, Winzenberg T (2012). The daily patterns of time use for parents of children with complex needs: a systematic review. J Child Health Care.

[12] Anclair M (2017). Fears, Stress and Burnout in Parents of Children with Chronic Conditions (Doctoral Thesis).

[13] Pitch N, Shahil A, Mekhuri S, Ambreen M, Chu S, Keilty K, Cohen E, Orkin J, Amin R (2023). Caring for children with new medical technology at home: parental perspectives. BMJ Paediatr Open.

[14] (2017). THRIVE: The future of integrated health service planning for children and youth in the Champlain region. CHEO.

[15] Peter E, Spalding K, Kenny N, Conrad P, McKeever P, Macfarlane A (2007). Neither seen nor heard: children and homecare policy in Canada. Soc Sci Med.

[16] (2020). Children and youth with medical complexity in Canada. Canadian Institution for Health Information.

[17] Whiting M (2013). Impact, meaning and need for help and support: The experience of parents caring for children with disabilities, life-limiting/life-threatening illness or technology dependence. J Child Health Care.

[18] McCann D (2021). Parenting children with complex needs. J Child Health Care.

[19] Nageswaran S, Golden S (2017). Factors associated with stability of health nursing services for children with medical complexity. Home Healthc Now.

[20] Sobotka SA, Lynch E, Peek ME, Graham RJ (2020). Readmission drivers for children with medical complexity: Home nursing shortages cause health crises. Pediatr Pulmonol.

[21] Feudtner C, Nye R, Hill DL, Hall M, Hinds P, Johnston EE, Friebert S, Hays R, Kang TI, Wolfe J, Pediatric Palliative Care Research Network Shared DataResearch (PPCRN SHARE) Project Group (2021). Polysymptomatology in pediatric patients receiving palliative care based on parent-reported data. JAMA Netw Open.

[22] Prentice TM, Gillam L, Davis PG, Janvier A (2018). Always a burden? Healthcare providers' perspectives on moral distress. Arch Dis Child Fetal Neonatal Ed.

[23] Amar-Dolan L, Horn M, O’Connell B, Parsons S, Roussin C, Weinstock P, Graham Rj (2020). “This Is How Hard It Is”. Family experience of hospital-to-home transition with a tracheostomy. Annals ATS.

[24] Thode S (2021). Better pediatric policies are needed. Home Healthc Now.

[25] Libby R, Imaizumi S (2008). Guidelines for Pediatric Home Health Care (2nd Edition).

[26] Wolsky K (2014). Establishment of a standard definition of practice readiness: Academia's vs practices' registered nurses perceptions related to new graduate nurse competencies within Alberta, Canada (Doctoral Thesis).

[27] Berkow S, Virkstis K, Stewart J, Conway L (2009). Assessing new graduate nurse performance. Nurse Educ.

[28] Peters MDJ, Marnie C, Tricco AC, Pollock D, Munn Z, Alexander L, McInerney P, Godfrey CM, Khalil H (2020). Updated methodological guidance for the conduct of scoping reviews. JBI Evid Synth.

[29] Moher D, Shamseer L, Clarke M, Ghersi D, Liberati A, Petticrew M, Shekelle P, Stewart LA, PRISMA-P Group (2015). Preferred Reporting Items for Systematic Review and Meta-Analysis Protocols (PRISMA-P) 2015 statement. Syst Rev.

[30] Page M, McKenzie J, Bossuyt P, Boutron I, Hoffmann T, Mulrow C, Shamseer L, Tetzlaff J, Akl Elie A, Brennan Sue E, Chou Roger, Glanville Julie, Grimshaw Jeremy M, Hróbjartsson Asbjørn, Lalu Manoj M, Li Tianjing, Loder Elizabeth W, Mayo-Wilson Evan, McDonald Steve, McGuinness Luke A, Stewart Lesley A, Thomas James, Tricco Andrea C, Welch Vivian A, Whiting Penny, Moher David (2021). The PRISMA 2020 statement: an updated guideline for reporting systematic reviews. BMJ.

[31] Major-Kincade Terri, Kim Erin, Price Cameron A, Banerjee Ankona, Franklin Nikashia, Castañeda Almendárez Israel, Jump Jaime, Rubenstein Jared, Jarrell Jill Ann (2025). The use of biased language in the care of seriously ill children: A pilot study. Am J Hosp Palliat Care.

[32] Smith K E, Bambra C, Joyce K E, Perkins N, Hunter D J, Blenkinsopp E A (2009). Partners in health? A systematic review of the impact of organizational partnerships on public health outcomes in England between 1997 and 2008. J Public Health (Oxf).

[33] Alderwick Hugh, Hutchings Andrew, Briggs Adam, Mays Nicholas (2021). The impacts of collaboration between local health care and non-health care organizations and factors shaping how they work: a systematic review of reviews. BMC Public Health.

[34] Sultan Marium A, Miller Emily, Tikkanen Roosa Sofia, Singh Shalini, Kullu Arpana, Cometto Giorgio, Fitzpatrick Siobhan, Ajuebor Onyema, Gillon Nicholas, Edward Anbrasi, Moleman Youri P, Pandya Shivani, Park Inyeong, Shen Jung Yu, Yu Yefei, Perry Henry, Scott Kerry, Closser Svea (2025). Competency-based education and training for community health workers: a scoping review. BMC Health Serv Res.

